# Genetic Dissection and Functional Differentiation of *ALK*^*a*^ and *ALK*^*b*^, Two Natural Alleles of the *ALK*/*SSIIa* Gene, Responding to Low Gelatinization Temperature in Rice

**DOI:** 10.1186/s12284-020-00393-5

**Published:** 2020-06-11

**Authors:** Zhuanzhuan Chen, Yan Lu, Linhao Feng, Weizhuo Hao, Chuang Li, Yong Yang, Xiaolei Fan, Qianfeng Li, Changquan Zhang, Qiaoquan Liu

**Affiliations:** 1 State Key Laboratory of Hybrid Rice, Jiangsu Key Laboratory of Crop Genomics and Molecular Breeding, Jiangsu Key Laboratory of Crop Genetics and Physiology, Yangzhou University, Yangzhou, 225009 China; 2 Key Laboratory of Plant Functional Genomics of the Ministry of Education / Jiangsu Co-Innovation Center for Modern Production Technology of Grain Crops, College of Agriculture, Yangzhou University, Yangzhou, 225009 China

**Keywords:** *Oryza sativa* L., *ALK* gene, Gelatinization temperature, Allelic variation, Starch fine structure

## Abstract

**Background:**

*ALK* is the key gene controlling rice gelatinization temperature (GT), which is closely associated with the eating and cooking quality (ECQ) in rice (*Oryza sativa* L.). To date, at least three *ALK* alleles are thought to be responsible for the diversity of GT among rice cultivars. The *ALK*^*c*^*/SSIIa*^*i*^ allele with high activity of the soluble starch synthase IIa (SSIIa) controls high GT, but the accurate functional difference between *ALK*^*a*^ and *ALK*^*b*^ alleles, both controlling low GT, is not clearly elucidated. Thus, we generated rice near-isogenic lines (NILs) by introducing different *ALK* alleles into the *japonica* cultivar Nipponbare (Nip) to clarify the discrepant effects of the two low-GT *ALK* alleles.

**Results:**

The results showed that the function of two low-GT alleles (*ALK*^*a*^ and *ALK*^*b*^) was different, and a much lower GT was observed in NIL(*ALK*^*b*^) rice grains compared with that of Nip(*ALK*^*a*^). Moreover, the starches of NIL(*ALK*^*b*^) grains had a higher degree of branching, higher setback, consistence and higher cool pasting viscosity than those of Nip(*ALK*^*a*^). The lower expression level of *ALK*^*b*^, compared with *ALK*^*a*^, resulted in depleted intermediate chains and increased short chains of amylopectin, thus affected the thermal and pasting properties of NILs’ grains. Also, the data revealed both low-GT alleles were mainly found in temperate *japonica*, but more *ALK*^*b*^ was found in other subpopulations such as *indica* as compared to *ALK*^*a*^.

**Conclusions:**

Overall, all the results suggested that the function between two low-GT alleles was different, and the distribution of *ALK*^*b*^ was much wider than that of *ALK*^*a*^ among the subpopulations of cultivated rice.

## Background

Rice, as one of the most important crops worldwide, is the staple food for about 50% of the world’s population. Currently, considering the increase in grain yield and improved living conditions, consumers are paying more attention to grain quality than ever before. Eating and cooking quality (ECQ) is one of the most important evaluation indexes to appraise rice quality, which can be evaluated using several physic-chemical characteristics including the gelatinization temperature (GT). The GT is the critical temperature at which about 90% of the starch granules absorb enough heat to irreversibly swell in hot water, and start to lose their crystallinity and appear anisotropic (Cuevas et al. 2010).

The GT in rice has been extensively studied in terms of inheritance and environmental response, and the gelatinization property of rice grains is tightly linked to the enzymes involved in starch biosynthesis during endosperm development (Chun et al. 2015; Nakamura et al. 2005; Xu and Mo 1996). The GT value is a quantitative trait that displays continuous variation over a wide range of rice cultivars, and it is well known that *ALK*/*SSIIa*, near the *Wx* locus on chromosome 6, is the major locus responsible for rice GT (Bao et al. 2004; Fan et al. 2005; Gao et al. 2003; Umemoto et al. 2002). *ALK*/*SSIIa* encodes the soluble starch synthase IIa (SSIIa) in rice, which plays a specific role in the synthesis of the long B_1_ chains by elongating the short A and B_1_ chains of amylopectin in endosperm. Analysis of *ALK* allelic variation by using near-isogenic lines (NILs), in which a narrow genomic region around the *SSIIa* gene of the low-GT *japonica* cultivar Nipponbare was replaced by that of the high-GT *indica* cultivar Kasalath, proved the hypothesis that SSIIa is the key enzyme controlling GT in rice (Umemoto et al. 2004). Jiang et al. (2004) compared the SSIIa expression levels between endosperms of two rice cultivars possessing different gelatinization properties, and their data indicated that the amount of SSIIa protein was reduced in the cultivar that was easy to gelatinize in urea or alkali. In rice, lack of SSIIa enzyme activity resulted in S-type amylopectin that was enriched in short A-chains, whereas high SSIIa activity in wild type produced L-type amylopectin that contained an abundance of long B-chains (Nakamura et al. 2005). The rice *ss2a* mutant showed significantly reduced SSIIa activity and higher level of short chains of amylopectin (degree of polymerization (DP) ≤ 12) than those in the wild type (Miura et al. 2018), which agreed with data derived from peas and barley. Craig et al. (1998) reported that the DP of the A chains of amylopectin was reduced in pea embryo that lacked SSII activity. In a barley shrunken grain mutant, the loss of SSIIa activity resulted in a shortened amylopectin chain length distribution and a reduced GT, suggesting that amylopectin structure is a determinant of the starch GT (Morell et al. 2010).

**Fig. 1:**
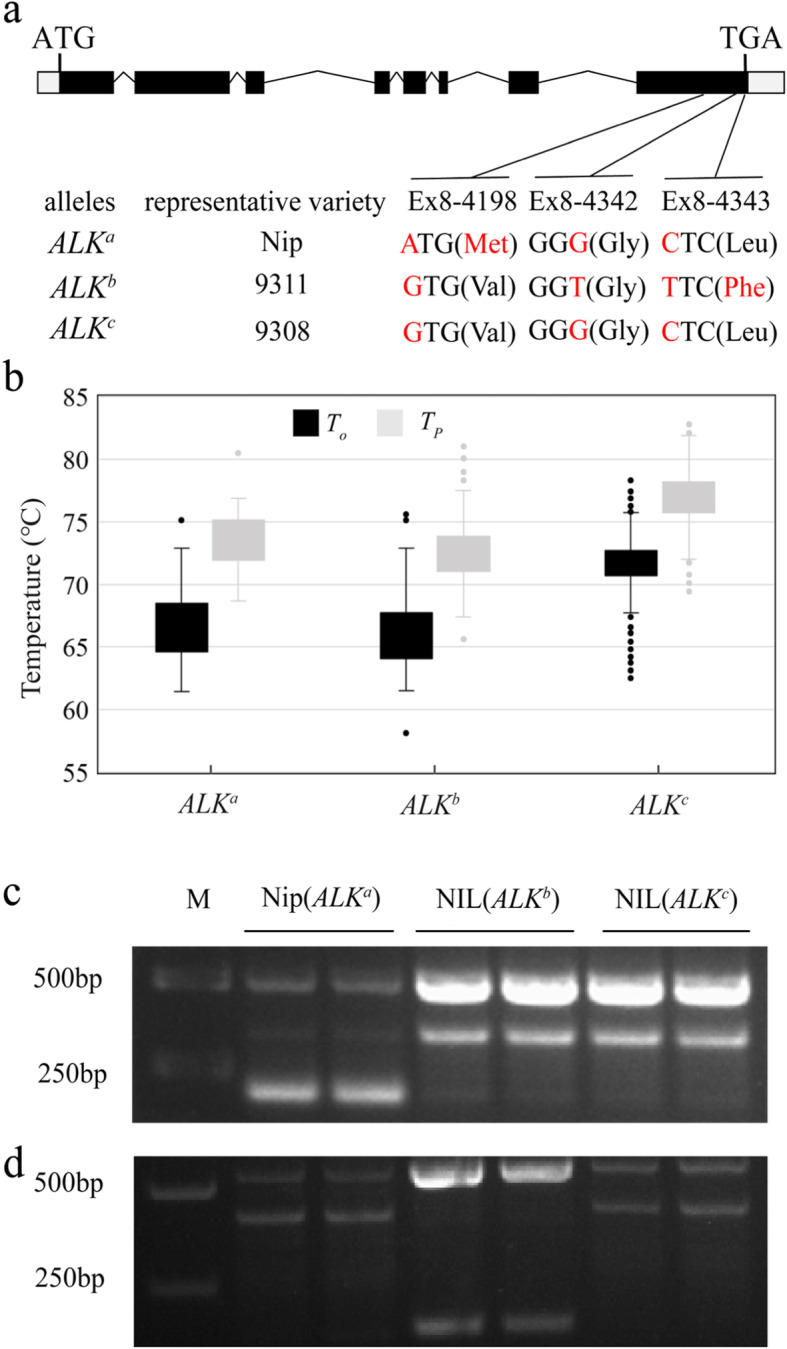
Natural variation and three main alleles of *ALK* gene in rice. **a** Schematic representation of *ALK* coding regions and its three main alleles classified by three single nucleotide polymorphisms (SNPs, shown in red letters) in exon 8. The start (ATG) and stop (TGA) codons were shown. Nip represents the rice cultivar Nipponbare. **b** The average gelatinization temperatures of rice grains with different *ALK* alleles among 399 rice accessions. *T*_*o*_ and *T*_*p*_ were the onset and peak temperatures during gelatinization measured by differential scanning calorimeter (DSC), respectively. **c** and **d** Confirmation of near-isogenic lines (NILs) carrying different *ALK* alleles by two functional molecular markers 4211 (G/A) (**c**) and 4342 (GC/TT) (**d**) by using the allele-specific PCR (AS-PCR), respectively

Several studies reported that at least three single nucleotide polymorphisms (SNPs) of the *ALK* gene are associated with the diversity of GT in rice (Bao et al. 2006; Nakamura et al. 2005; Umemoto et al. 2008; Waters et al. 2006). Nakamura et al. (2005) further analyzed the effect of amino acid substitutions caused by these SNPs on SSIIa enzyme activity, and suggested that two of the SNPs were associated with approximately 10% reduction of SSIIa activity in *japonica* compared with that in *indica*. Taken together, three SNPs in exon 8 of rice *ALK* gene, at positions 4211 bp (G/A), 4342 bp (G/T) and 4343 bp (C/T), respectively, downstream of the ATG start codon (Fig. 1a), were proven to be tightly associated with variation in alkali starch disintegration. Based on these functional nucleotide polymorphisms (FNPs), there are at least three *ALK* haplotypes among rice cultivars, namely A-GC, G-TT, and G-GC, with the G-GC haplotype being predominant (Bao et al. 2006; Gao et al. 2011; Tian et al. 2009; Waters et al. 2006). They were classified into two types based on GT level, the active *indica* type *SSIIa*^*i*^ (G-GC) controlling a high GT and inactive *japonica* types *SSIIa*^*j*^ (A-GC) or (G-TT) controlling a low GT (Gao et al. 2011; Luo et al. 2015; Shimbata et al. 2012). Recently, Zhou et al. (2016) analyzed the nucleotide diversity and molecular evolution of the *ALK* gene in cultivated rice and their wild relatives, and suggested that the A-GC and G-TT haplotypes of *ALK* locus may be derived from the G-GC haplotype. Even now, the accurate GT and chain length distribution of amylopectin are not unique to all the haplotypes, and it is likely that the genetic effects of natural variation in the rice *ALK/SSIIa* gene has not been fully determined (Umemoto et al. 2008).

To date, even though three haplotypes of the *ALK* locus have been identified among rice cultivars, only the differences caused by the *SSIIa*^*i*^ and *SSIIa*^*j*^ types have been carefully compared in most studies (Gao et al. 2011; Inukai 2017; Luo et al. 2015; Shimbata et al. 2012). These studies rarely discussed the similarities and/or differences between the two haplotypes, A-GC and G-TT, which were grouped as the same *SSIIa*^*j*^ type, both controlling low GT. Moreover, although the expression levels and enzyme activities of various *ALK* alleles were compared among different cultivars (Nakamura et al. 2005), studies rarely focused on the effects of these haplotypes in the same genetic background, such as that found in NILs. In the present study, by association analysis between the GT and *ALK* genomic sequence among rice cultivars, we confirmed that the aforementioned three SNPs in *ALK* are essential for the GT diversity in rice cultivars. These SNPs make up the three haplotypes/alleles described in this study, the two low-GT alleles are identified as *ALK*^*a*^ (A-GC) and *ALK*^*b*^ (G-TT), respectively, while the high-GT allele is identified as *ALK*^*c*^ (G-GC). To delineate the relationship between these alleles, we generated two NILs, NIL(*ALK*^*b*^) and NIL(*ALK*^*c*^), by introgressing the *ALK*^*b*^ (G-TT) and *ALK*^*c*^ (G-GC) alleles from the *indica* cultivars, 9311 and 9308, respectively, into a Nipponbare background. Nipponbare has the *ALK*^*a*^ allele, hereafter referred to as Nip(*ALK*^*a*^). Using NIL(*ALK*^*c*^), exhibiting high GT, as the control, differences between the allelic function of *ALK*^*a*^ and *ALK*^*b*^, both exhibiting low GT, were elucidated in the same *japonica* background.

## Results

### Confirmation of Three Main Alleles of *ALK* Gene in Rice Accessions

Based on the alignments among the *ALK* genomic sequences of 399 rice cultivars originating from rice growing regions around the world, we identified 40 SNPs and 12 insertion/deletions (InDels) in introns, and 23 SNPs and 13 InDels in exons (Additional file 1: Figure S1). Association analysis between grain GT and the above polymorphisms of all the rice cultivars mentioned, showed that the GT variation in selected 399 rice accessions was mainly related to three SNPs in exon 8, Ex8–4211 (G/A), Ex8–4342 (G/T) and Ex8–4343 (C/T) (Fig. 1a-b; Additional file 1: Figure S1). The SNP at Ex8–4342 (G/T) is a synonymous mutation. The Ex8–4211 (G/A) and Ex8–4343 (C/T) SNPs cause two amino acid substitutions in SSIIa enzyme, valine (Val) to methionine (Met) on 737th residue and leucine (Leu) to phenylalanine (Phe) on 781st residue, respectively. Combinations of these FNPs resulted in three *ALK* haplotypes, A-GC (*ALK*^*a*^), G-TT (*ALK*^*b*^) and G-GC (*ALK*^*c*^) in rice cultivars (Fig. 1a), which was consistent with previous studies (Gao et al. 2011; Zhou et al. 2016). Among them, the A-GC (*ALK*^*a*^) and G-TT (*ALK*^*b*^) haplotypes appeared to have low (73.28 °C of *T*_p_) and very low (72.57 °C of *T*_p_) GT, respectively, while the G-GC (*ALK*^*c*^) haplotype contributed to a high (76.94 °C of *T*_p_) GT (Fig. 1b). Therefore, two reliable allele-specific PCR (AS-PCR) molecular markers (Additional file 2: Table S1; Fig. 1c-d) were developed based on the three SNPs for classification of *ALK* haplotypes in other 41 rice cultivars (Additional file 2: Table S2). Among these 440 rice cultivars (Additional file 2: Table S2 and S3), the two haplotypes for low-GT alleles were found mainly in temperate *japonica*, but more *ALK*^*b*^ was found in *indica* than *ALK*^*a*^. While the *ALK*^*c*^ (G-GC) for high-GT was mostly found in *indica* accessions, confirming previous studies (Nakamura et al. 2005; Gao et al. 2011; Zhou et al. 2016).

### Functional Differentiation between Low-GT Alleles, *ALK*^*a*^ and *ALK*^*b*^

With the aim to compare the accurate function between two low-GT alleles, *ALK*^*a*^ and *ALK*^*b*^, in cultivated rice, we generated the NIL (NIL(*ALK*^*b*^)) by introgressing the low-GT allele *ALK*^*b*^ from *indica* cultivar 9311 into the *japonica* Nipponbare background (Additional file 1: Figure S2a). Nipponbare (Nip) carrying the low-GT *ALK*^*a*^ allele was named as Nip(*ALK*^*a*^) here. Meanwhile, another NIL, NIL(*ALK*^*c*^) with the high-GT allele *ALK*^*c*^ from *indica* cultivar 9308 was also obtained at the same time. The two NILs had the same morphology as Nip plants in the paddy field (Additional file 1: Figure S2b). Also, whole genome sequencing did not reveal any background introgressions in the two NILs except the targeted segments from the donor 9311 (*ALK*^*b*^) or 9308 (*ALK*^*c*^) (Additional file 1: Figure S3).

**Fig. 2:**
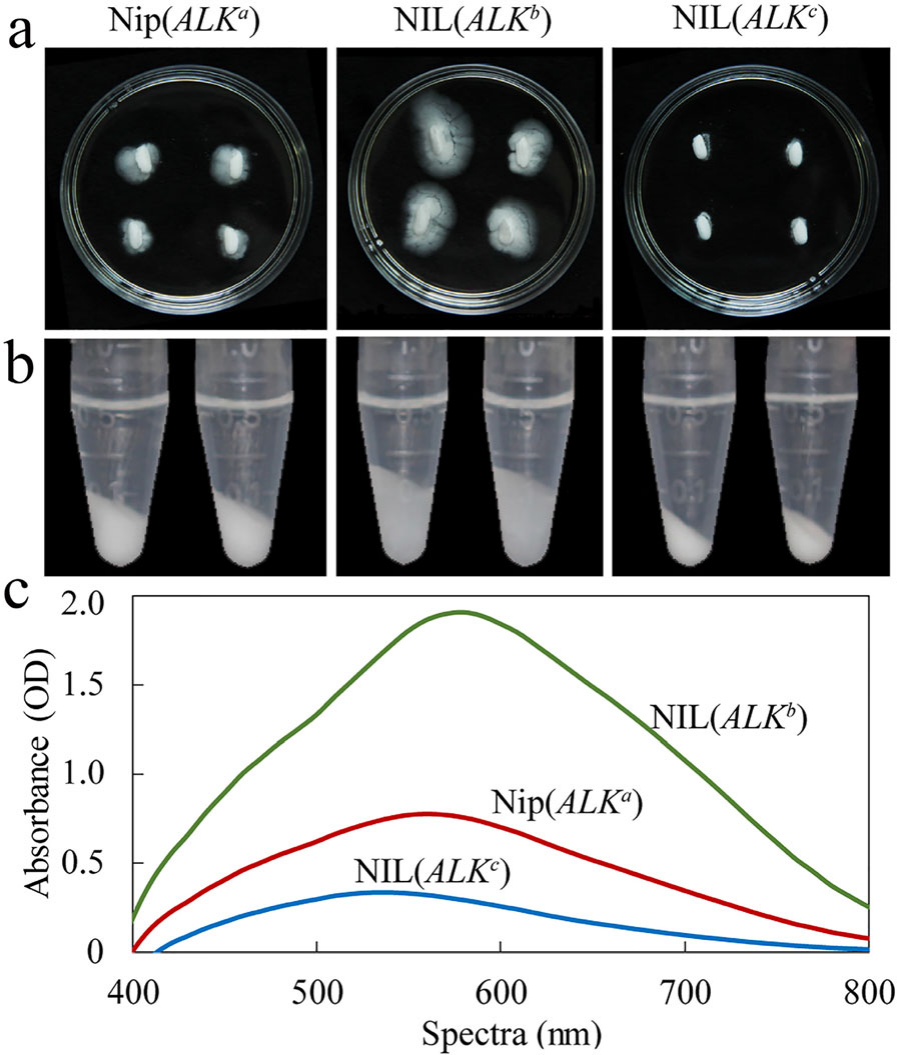
Gelatinization properties of rice grains or flours from three near-isogenic lines (NILs) with different *ALK* alleles. **a** Alkali spreading test of milled rice in 1.4% potassium hydroxide (KOH) solution. **b** Gelatinization of rice flour in 4.0 mol/L urea solution. **c** I_2_-KI absorption spectroscopic analysis of the gelatinized supernatant fractions from rice flours in urea solution as shown in panel **b**

**Fig. 3:**
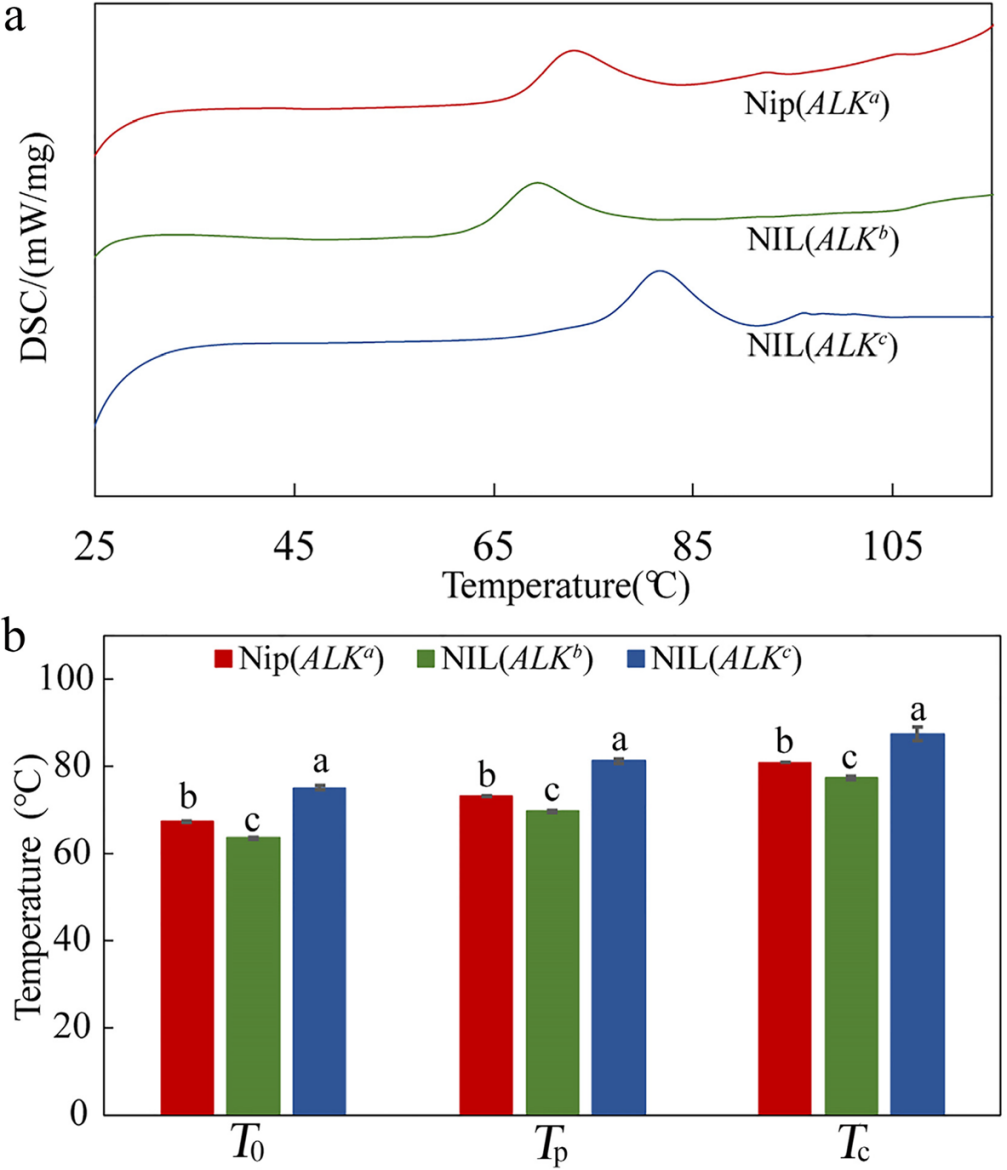
Thermal properties of rice flours from near-isogenic lines (NILs) with different *ALK* alleles detected by differential scanning calorimeter (DSC). **a** DSC curves. **b** The peak (*T*_o_), onset (*T*_p_) and terminating (*T*_c_) temperatures during gelatinization measured by differential scanning calorimeter (DSC). Error bars are mean ± s.d. (*n* = 3), and the small letters above error bars indicate significant differences among the three NILs (*P* < 0.05)

The results of the alkali spreading test (Fig. 2a) indicated both Nip(*ALK*^*a*^) and NIL(*ALK*^*b*^) kernels showed a higher degree of gelatinization, thus lower GT, than NIL(*ALK*^*c*^). But the NIL(*ALK*^*b*^) grains had the highest degree of gelatinization, and Nip(*ALK*^*a*^) showed a slightly lower degree of gelatinization than NIL(*ALK*^*b*^). Additionally, we used differential scanning calorimetry (DSC) to determine the effects of *ALK* alleles on the grain physicochemical properties (Fig. 3). DSC showed Nip(*ALK*^*a*^) rice flour had a higher GT compared to NIL(*ALK*^*b*^) flour, thus the alkali spreading, gelatinization in urea and DSC analyses confirmed the significantly higher GT observed in Nip(*ALK*^*a*^) grains compared to NIL(*ALK*^*b*^) grains.

Previous studies showed the solubility of starch in organic solvents, such as dimethyl sulfoxide and urea, could also reflect the gelatinization properties of starch (Adkins et al. 1970; Baba et al. 1983; Fu and Xue 2010; Nishi et al. 2001). As shown in Fig. 2b, in urea solvents, the expansion volume of rice flour from Nip(*ALK*^*a*^) was less than the expansion of NIL(*ALK*^*b*^). Furthermore, we analyzed the binding ability by iodine solution of starch in the above urea solution. The peak of absorption curve of Nip(*ALK*^*a*^) was much lower than NIL(*ALK*^*b*^), showing an absorbance peak at 550 nm, while the NIL(*ALK*^*b*^) had the highest peak at 580 nm (Fig. 2c). These results further confirmed the functional differences between the two alleles *ALK*^*a*^ and *ALK*^*b*^ in rice.

**Fig. 4:**
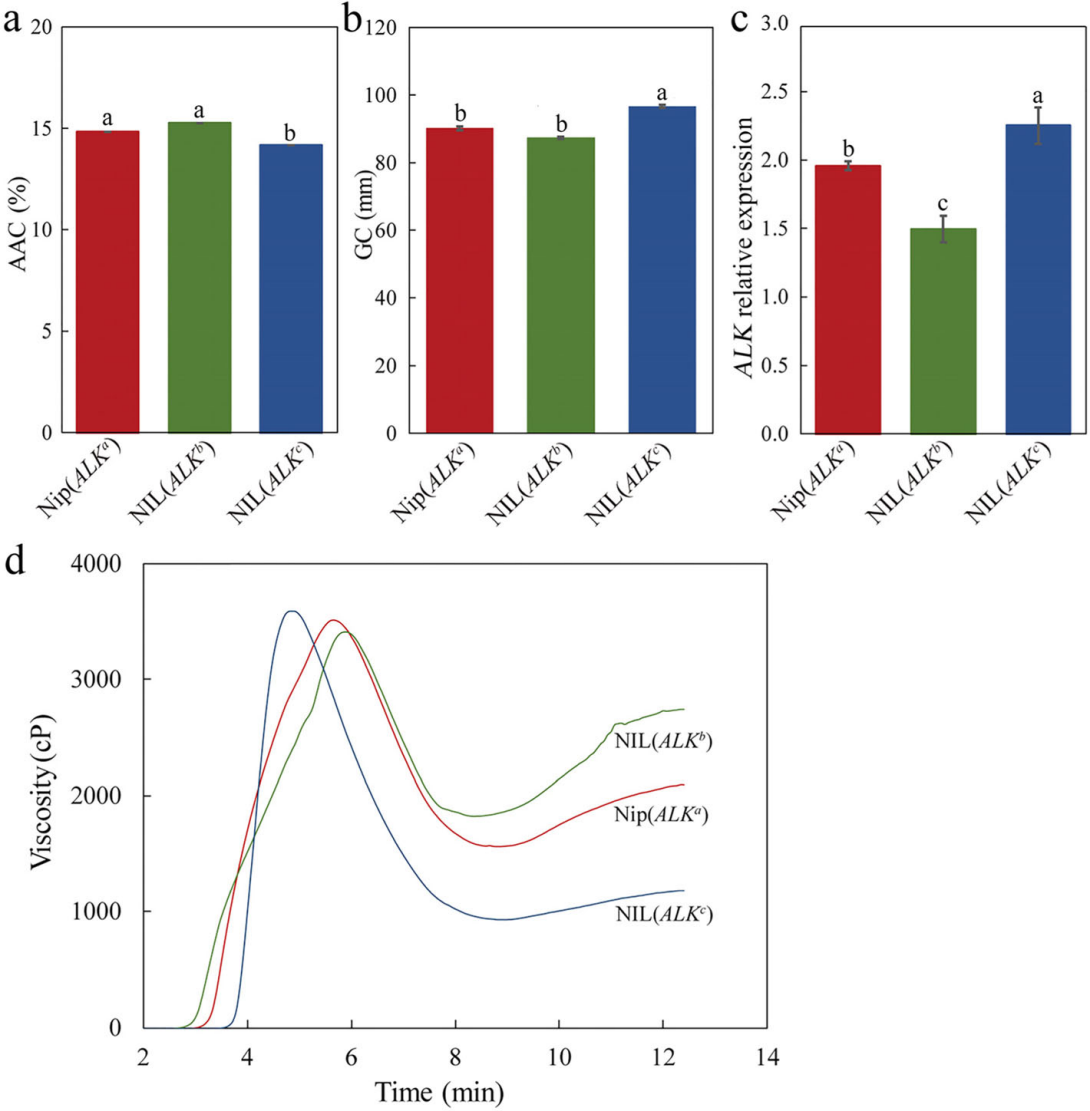
Physicochemical characteristics of mature grains from different near-isogenic lines (NILs) and expression levels of different *ALK* alleles in developing seed. **a** Apparent amylose content (AAC) in mature rice grains. **b** Gel consistency (GC) in mature rice grains. **c** Transcriptional levels of *ALK* gene in developing seeds at 15 days after flowering (DAF). **d** Rapid Visco Analyzer (RVA) curves of rice flours from mature rice grains. Error bars are mean ± s.d. (n = 3), and the small letters above error bars indicate significant differences among subpopulations (*P* < 0.05)

Both Nip(*ALK*^*a*^) and NIL(*ALK*^*b*^) grains had comparable AAC (apparent amylose content) and GC (gel consistency) (Fig. 4a-b), the other two important determinants of rice ECQ (Tian et al. 2009). The high-GT NIL(*ALK*^*c*^) grains showed slightly lower AAC and harder GC than the two low-GT alleles (Fig. 4a-b), consistent with some previous studies (Miura et al. 2018). Taken together, the significant difference in gelatinization characters between Nip(*ALK*^*a*^) and NIL(*ALK*^*b*^) might be caused by differences in the amylopectin fine structure contributed by the different *ALK* alleles, *ALK*^*a*^ and *ALK*^*b*^.

### Expression Comparison between *ALK*^*a*^ and *ALK*^*b*^

*ALK* is expressed specifically in rice endosperm at the mid to relatively later stages of grain filling (Li et al. 2013; Hirose and Terao 2004). Therefore, total RNAs from NILs’ endosperms at 15 days after flowering (DAF) were extracted for expression analysis of the *ALK* gene. The data showed that the *ALK* transcription in the NIL(*ALK*^*b*^) endosperms were significantly lower than that in Nip(*ALK*^*a*^) (Fig. 4c), which reflected in the subtle difference of GT between the two low-GT lines (Fig. 3b). Besides, the difference of expression level of the *ALK* gene between NIL(*ALK*^*c*^) and NIL(*ALK*^*a*^) was larger than that between the two low GT lines (Fig. 4c), which was consistent with the much higher GT in NIL(*ALK*^*c*^). This implies the relative transcriptional expression of three *ALK* alleles is correlated with GT. In other words, the higher the expression level of the *ALK* gene, the higher GT in the rice samples and suggests the discrepant effects on the GT might be partly caused by the differential expression level of *ALK* in the three NILs.

### Difference of Amylopectin Fine Structure between Nip(*ALK*^*a*^) and NIL(*ALK*^*b*^)

**Fig. 5:**
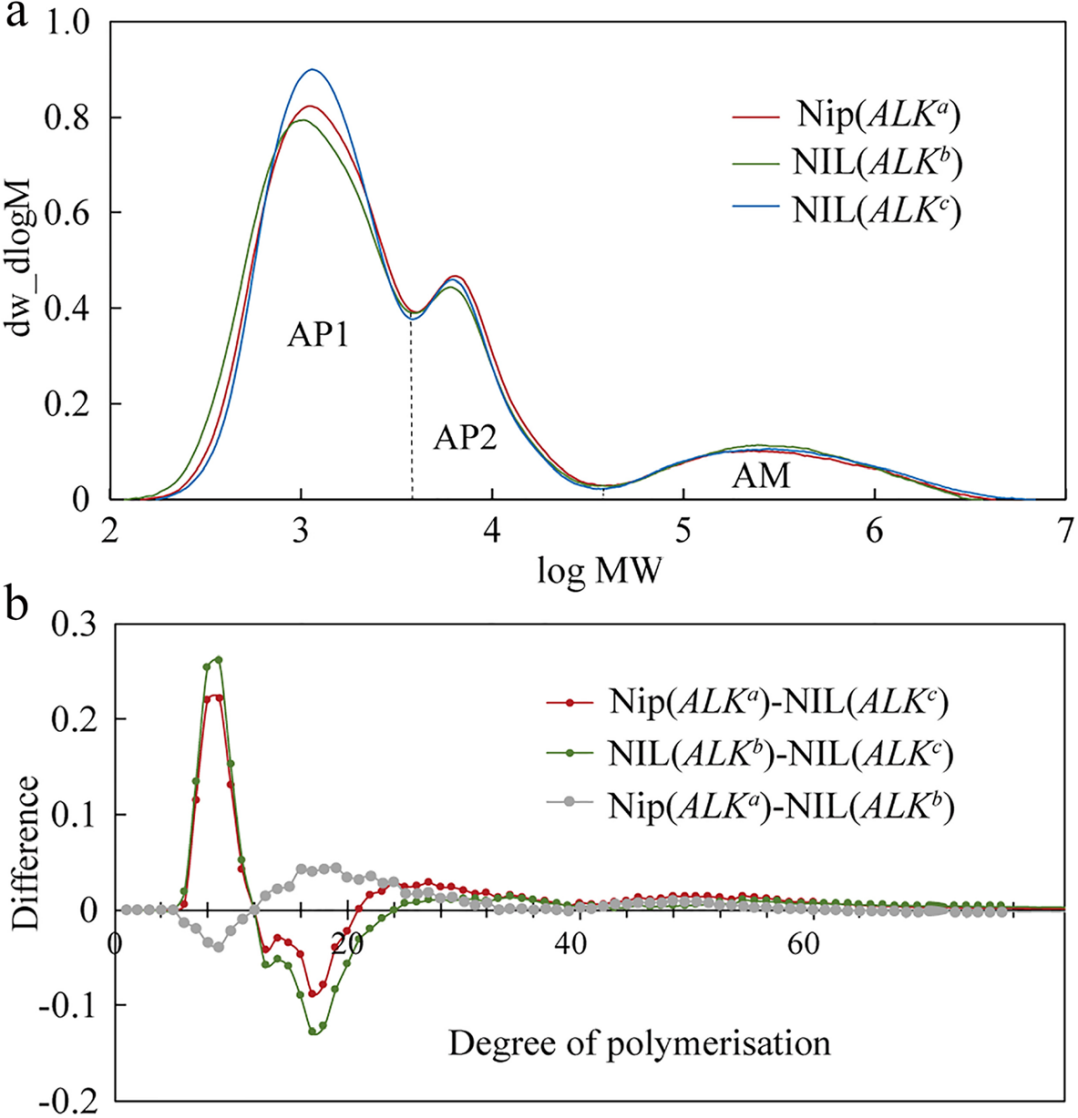
Comparison of starch fine structures among rice grains from different near-isogenic lines (NILs). **a** Gel permeation chromatography (GPC) curves of debranched starch from NILs’ mature seeds. AP1 and AP2 represent the short chain (AP1) and long chain (AP2) fractions of amylopectin, respectively. AM means the amylose fraction. **b** The difference of degree of polymerization (DP) of debranched starch of different NILs

The amylopectin (AP) detected by gel permeation chromatography (GPC) could be divided to short chain (AP1) and long chain (AP2) fractions (Fig. 5a), which mainly comprise low molecular weight molecules such as A and short B chains (A + B_1_ chains), and long B chains with higher molecular weight, respectively (Wang et al. 2015). The ratio of AP1 to AP2 fractions is usually used as an index of the extent of amylopectin branching: the higher the ratio, the higher the degree of starch branching. As shown in Fig. 5a and Table 1, the relative amount of the AP1 fraction of NIL(*ALK*^*b*^) (63.59%) was slightly higher than that of Nip(*ALK*^*a*^) (63.15%), although there was no significant difference among the three starch samples. By contrast, Nip(*ALK*^*a*^) had a significantly higher AP2 level (23.47%) compared to the AP2 level for NIL(*ALK*^*b*^) (21.72%) and NIL(*ALK*^*c*^) (22.56%). The data in Table 1 also indicated that the amylopectin of NIL(*ALK*^*b*^) showed the highest degree of branching (AP1/AP2 = 2.93), suggesting that it might generate more A and short B_1_ chains than another low GT line Nip(*ALK*^*a*^) (AP1/AP2 = 2.69). Besides, the GPC data revealed that there was no significant difference in the levels of true amylose (AM) among three NILs, though a relatively low amylose content was found in NIL(*ALK*^*c*^) starch (Table 1).

**Table 1:** Gel permeation chromatography (GPC) parameters, relative crystallinities and infrared ratios (IR) among near-isogenic lines (NILs) ^a^

Genotype	AP1 (%)	AP2 (%)	AP1/AP2	AM (%)	Relative crystallinity (%)	1047/1022 (cm^−1^)
Nip(*ALK*^*a*^)	63.15 ± 0.50a	23.47 ± 0.10a	2.69 ± 0.03b	13.37 ± 0.50a	31.7 ± 0.21b	0.389 ± 0.001b
NIL(*ALK*^*b*^)	63.59 ± 1.20a	21.72 ± 0.50c	2.93 ± 0.07a	14.01 ± 0.70a	35.1 ± 0.14a	0.382 ± 0.002b
NIL(*ALK*^*c*^)	64.18 ± 0.50a	22.56 ± 0.30b	2.85 ± 0.03a	13.27 ± 0.70a	35.9 ± 0.14a	0.397 ± 0.001a

^a^Data represent means ± standard deviations, *n* = 2. AP1, AP2, and AP1/AP2 represent the relative fractions correspond to short (AP1) and long (AP2) branch chains of amylopectin, and the degree of amylopectin branching (AP1/AP2), respectively. AM represents the true amylose content (AM) as determined by gel permeation chromatography (GPC) parameters. The relative crystallinity was calculated from XRD. Ratios of 1047/1022 represents the amount of ordered starch calculated from ATR-FTIR. The small letters in each column indicate significant differences among genotypes (*P* < 0.05)

Moreover, we further analyzed the degree of polymerization (DP) of amylopectin fractions using high-performance anion-exchange chromatography (HPAEC) approach. As shown in Fig. 5b, NIL(*ALK*^*b*^) exhibited significantly more short chains (DP6–12) and obviously fewer intermediate chains (DP13–21) compared with Nip(*ALK*^*a*^). The results could also explain the GT variation between the two low-GT NILs (Fig. 3), because it is clear that a decrease of the amylopectin chains with DP < 10 could increase rice GT (Umemoto et al. 2004; Umemoto et al. 2008). These data were consistent with previous studies that different *ALK* alleles could alter the amylopectin structure, in which intermediate chains (DP12–24) were enriched, and short chains (DP < 11) were depleted in *indica* cultivars compared with those in *japonica* cultivars (Miura et al. 2018; Umemoto et al. 2008; Umemoto et al. 2002).

### Comparison of Pasting Properties between Nip(*ALK*^*a*^) and NIL(*ALK*^*b*^)

The pasting properties of rice grains could be evaluated using the RVA profiles and the characteristics peak viscosity (PKV), hot peak viscosity (HPV), breakdown (BDV), setback (SBV), consistence viscosity (CSV), cool pasting viscosity (CPV), peak time (PeT) and pasting temperature (PaT) (Zhang et al. 2013). Previous studies showed that the RVA these characteristics had a significant association with variations of the *ALK* alleles (Asante et al. 2013). As shown in Fig. 4d and Table 2, rice starch from mature seeds of the three NILs exhibited very different pasting properties. NIL(*ALK*^*b*^) starch showed a higher value of final viscosity, CPV, than that of Nip(*ALK*^*a*^) at the end of the RVA curve (Fig. 4d). Also, there were slightly differences between the two low-GT NILs, for three RVA characteristics where NIL(*ALK*^*b*^) has a higher SBV, CSV, and CPV than Nip(*ALK*^*a*^) (Table 2).

**Table 2:** Comparison of the pasting properties of rice starch among near-isogenic lines (NILs) ^a^

Genotype	PKV (cP)	HPV (cP)	BDV (cP)	CPV (cP)	SBV (cP)	CSV (cP)	PeT (min)	PaT (°C)
Nip(*ALK*^*a*^)	3516 ± 11a	1562 ± 18 b	1954 ± 7b	2096 ± 18b	− 1420 ± 5b	534 ± 12b	5.7 ± 0.0b	76.65 ± 0.01b
NIL(*ALK*^*b*^)	3407 ± 7b	1820 ± 10a	1587 ± 13c	2739 ± 11a	−668 ± 8a	919 ± 23a	5.9 ± 0.0a	73.45 ± 00.02c
NIL(*ALK*^*c*^)	3587 ± 9a	925 ± 25c	2662 ± 8a	1177 ± 7c	− 2410 ± 6c	252 ± 8c	4.9 ± 0.0c	82.25 ± 00.01a

^a^Data represent means ± standard deviations, n = 2. *PKV* Peak viscosity, *HPV* Hot peak viscosity, *BDV* Breakdown, *CPV* Cool pasting viscosity, *SBV* Setback, *CSV* Consistence viscosity, *PeT* Peak time, *PaT* Pasting temperature. The small letters in each column indicate significant differences among genotypes (*P* < 0.05)

## Discussion

### Differences in NIL Gelatinization Properties Caused by *ALK* Allelic Variation

Several studies have been performed to dissect the function of the different *ALK/SSIIa* alleles found in different rice cultivars (Bao et al. 2006; Nakamura et al. 2005; Umemoto et al. 2008; Waters et al. 2006). In this study, we used NILs, containing three different *ALK* alleles, introgressed into the background of the temperate *japonica* rice cultivar, Nipponbare, the recurrent parent, thus all other starch synthesis-related genes were the same across the three NILs and the difference among the gelatinization properties would obviously be caused by the allelic variation of *ALK* locus, agreeing with previous studies (Bao et al. 2006; Gao et al. 2011; Umemoto et al. 2008). The allelic variations were mainly caused by the three SNPs in the *ALK* coding sequence, as the SNPs Ex8–4211 (G/A), Ex8–4342 (G/T) and Ex8–4343 (C/T) in exon 8 (Fig. 1a), which showed the highest association with GT diversity in different cultivars (Additional file 1: Figure S1). Where, the cultivars or NIL with a combination of G-GC (*ALK*^*c*^), meaning Ex8–4211 (G), Ex8–4342 (G) and Ex8–4343 (C), appeared to have a dramatically higher GT than those with other combinations (Figs. 1 and 2). Most importantly, the SNPs Ex8–4211 (G/A) and Ex8–4343 (C/T) caused two residue replacements in the encoded SSIIa enzyme, Val to Met in the 737th residue and Leu to Phe in 781st residue (Fig. 1a), respectively. Both residues at the corresponding sites are located in the predicted glucose transfer domain (residues 610–785) and thus are essential for SSIIa function (Nakamura et al. 2005). It implied that the replacements of either Val by Met (such as in *ALK*^*a*^) or Leu by Phe (such as in *ALK*^*b*^) resulted in a low or very low GT. In addition, our data showed that the GT controlled by *ALK*^*b*^ was relatively lower than that of *ALK*^*a*^, therefore, replacing Leu with Phe, like *ALK*^*c*^ to *ALK*^*b*^, decreased the GT much more than replacing Val with Met, like *ALK*^*c*^ to *ALK*^*a*^.

### Distribution of *ALK*^*a*^ and *ALK*^*b*^ in Rice Subpopulations

In the present study, we analyzed the distribution of the three *ALK* alleles in 440 rice accessions representing the five rice subpopulations (*indica*, *aus*, *aromatic*, temperate *japonica* and tropical *japonica*) including accessions which were admixtures or unclassified (Additional file 2: Table S2 and S3). A total of 267 accessions were found to carry the *ALK*^*c*^ (G-GC) allele, which was found in all subpopulations, but predominantly in *indica* varietal group (comprised of the *indica* and *aus* subpopulations, 180/267, 67.42%) and tropical *japonica* (TRJ) group (Additional file 2: Table S3). In the case of 65 accessions with the *ALK*^*a*^ (A-GC) allele, most (47/65, 72.31%) were temperate *japonica* (TEJ) and only 6 (9.23%) were *indica*. Interestingly, the distribution of *ALK*^*b*^ (G-TT) is much wider than *ALK*^*a*^ (A-GC). Among 108 samples with *ALK*^*b*^ (G-TT), 56 (51.85%) were TEJ while 23 (23.30%) were *indica*. More interestingly, when compared the distribution of the three alleles between the two *japonica* subpopulations, TRJ and TEJ, we found that *ALK*^*c*^ mainly exists in TRJ (48/53), while *ALK*^*a*^ (47/55) and *ALK*^*b*^ (56/63) are mainly in TEJ (Additional file 2: Table S3). Therefore, as the favorable *ALK*^*b*^ allele has a wide distribution in both *indica* and *japonica*, it should be easy to introgress into either *indica* or *japonica* cultivars to improve grain quality by using MAS. Previously, the *ALK* sequence analysis of 199 wild rice accessions by Zhou et al. (2016) revealed the G-GC haplotype (*ALK*^*c*^ allele) mainly existed in wild rice, but both A-GC (*ALK*^*a*^) and G-TT (*ALK*^*b*^) haplotypes were found in some wild rice accessions at a very low frequency. Thus, based on the wide distribution of *ALK*^*c*^ allele in both wild rice and all subpopulations of cultivated rice, it is implied that *ALK*^*c*^ might be the ancestral allele of *ALK* locus, and both two low-GT alleles (*ALK*^*b*^ and *ALK*^*a*^) might have evolved from *ALK*^*c*^ during the early stages of rice domestication as suggested by Zhou et al. (2016) and Singh et al. (2017).

### *ALK*^*b*^ Controls a Lower GT than *ALK*^*a*^ Due to the Lower Expression Level of *ALK*

Rice GT is positively associated with the expression level of *ALK* in the endosperm (Gao et al. 2011; Crofts et al. 2017), and it was reported that amino acid replacements among different *ALK* alleles also could change the SSIIa enzyme activity, as well as its binding affinity to starch granules (Nakamura et al. 2005; Crofts et al. 2017; Miura et al. 2018). Nakamura et al. (2005) found that Val-737 and Leu-781 are essential not only for the optimal SSIIa activity, but also the capacity to synthesize *indica*-type amylopectin. In the present study, the peak temperature (*T*_p_) of DSC curve of NIL(*ALK*^*b*^) grains was 3.4 °C lower than that of Nip(*ALK*^*a*^) and this difference was, at least partly, due to the altered expression level of *ALK* during the milk stage (Fig. 4c). More recently, Miura et al. (2018) found that the *ss2a* (*alk*) null mutation in the EM204 line could reduce the GT by 5.6 °C compared with that of wild type carrying the *ALK*^*a*^ allele. Therefore, it is implied that the SSIIa enzyme in *ALK*^*b*^ endosperm might still have some activity and considering the low transcription level and amino acid substitution in *ALK*^*b*^, we supposed the very low GT in *ALK*^*b*^ rice is regulated by both the enzymatic activity level and the transcriptional level of SSIIa during endosperm development.

### Alteration in Fine Structure of Amylopectin Caused by Different *ALK* Alleles

It is well known that SSIIa plays a distinct role in the elongation of short chains within a cluster (A + B_1_ chains) of amylopectin in the cereal endosperm, and this role cannot be complemented by other soluble starch synthase isoforms. The *ss2a*-deficient mutants from wheat (Yamamori et al. 2000), barley (Morell et al. 2010) and maize (Takeda and Preiss 1993) had a modified amylopectin structure with enriched short chains, which altered the physicochemical properties of the starch granules.

Amylose is synthesized by GBSSI encoded by the *Wx* gene and the amylose content (AC) correlates positively with the level of GBSSI in rice endosperm (Zhang et al. 2019). As *Wx* displays genetic linkage to the *ALK* locus on the short arm of chromosome 6 in rice, it is hard to dissect the relationship between GT and AC by using different rice cultivars, but the correlation between GT and AC was found to be significantly negative or null (Nakamura et al. 2002). Gao et al. (2011) found that, though there was no correlation between GT and AC among different rice cultivars, the AC decreased significantly in transgenic Nipponbare lines for *ALK* with high GT. In a recent report, Miura et al. (2018) showed the AC in a rice *ss2a*-deficient mutant with a low GT was higher than that in the wild type (Kinmaze) with high GT. Similarly, we found that the AAC of both NIL(*ALK*^*a*^) and NIL(*ALK*^*b*^) grains with low GT was higher than that of the high-GT NIL(*ALK*^*c*^) (Fig. 4a).

In the present study, both GPC and HPAEC analyses showed that NIL(*ALK*^*b*^) exhibited significantly higher amounts of short chains (DP 6–12) and markedly lower amounts of intermediate chains (DP 13–21) compared with Nip(*ALK*^*a*^). The proportion of DP > 10 in amylopectin in NIL(*ALK*^*b*^) was related to higher gelatinization by forming double helices, which are thought to be responsible for starch crystallinity (Srichuwong et al. 2005). Thus, we further measured the starch crystalline structure using both X-ray Diffraction (XRD) and Attenuated Total Reflectance-Fourier Transform Infrared System (ATR-FTIR) methods. These analyses revealed the three starch samples in NILs showed a similar XRD and FTIR spectra patterns (Additional file 1: Figure S4) but NIL(*ALK*^*b*^) showed a higher relative crystallinity (35.1%) than that of Nip(*ALK*^*a*^) (31.7%) (Table 1), and the ratio of FTIR absorbance values at 1047 and 1022 cm^− 1^ (1047/1022) showed similar results among the three NILs (Table 1). Because the GPC data showed no significant change in the true amylose (AM) level among the three NILs (Table 1), we suppose the low crystallinity in Nip(*ALK*^*a*^) starch might be caused by the difference in a higher proportion of amylopectin DP ≥ 22 compared with that in NIL(*ALK*^*b*^). The above results indicate the DSC parameters (*T*_o_, *T*_p_ and *T*_c_) are influenced by the molecular architecture of the crystalline region of starch, which corresponds to the distribution of short-chain amylopectin (DP6–11).

### *ALK* Allelic Variation Causes Difference of Not Only GT, but Also Pasting Properties

As there were no significant differences for AAC (Fig. 4a) and true amylose level (Table 1) between the two low-GT NILs, which carried the same *Wx*^*b*^ allele, it obviously implied that the alteration of pasting properties of our NILs (Fig. 4d) might have been caused by the different DPs of amylopectin controlled by the *ALK* allelic variation. Wang et al. (2016) reported that if the amylopectin contains more long chains, it may form more double helix structures or complexes with other components of rice, such as lipids, which can inhibit the swelling of starch and lead to a decrease in maximum viscosity. During RVA analysis, when starch gelatinization reaches its maximum viscosity, the swollen starch granules break down, and the amylose in the starch will flow out. If the amylopectin contains more long chains, it is difficult to break the structure of amylopectin. It is better to maintain the structure of starch granules during gel thickening, at which point the hot pasting viscosity will increase but the setback value will decrease because the setback value is derived from the peak viscosity minus the heat paste viscosity. Considering that other components in rice grains, such as protein particles and lipids, also effect the gelatinization of rice flour, it will be difficult to accurately elucidate the relationship between the chain length distribution of amylopectin and other RVA characteristics but considering the higher content of short chains in amylopectin favours gelatinization of starch grains, this fact might explain the lower peak viscosity but higher setback value in the low-GT line NIL(*ALK*^*b*^).

## Conclusions

The lower expression level of *ALK*^*b*^, compared with *ALK*^*a*^, may result in depleted intermediate chains and increased short chains of amylopectin and affect the thermal and pasting properties. In conclusion, the data supports the fact that the function of two low-GT alleles is different, and the distribution of *ALK*^*b*^ is much wider than that of *ALK*^*a*^ among rice subpopulations. These results give insight into the differentiation in ECQs caused by the natural allelic variation of *ALK* locus, and provide a reliable basis for breeding of high-quality rice.

## Methods

### Plant Materials

A total of 440 rice accessions were used in this study (Additional file 2: Table S2). Rice samples numbered 1 through 399 were sequenced in the region of the *ALK* gene, including *japonica* cultivar Nipponbare (Nip), 238 Rice Diversity Pane 1 (RDP1) (Eizenga et al. 2014) accessions (uppercase G), 109 rice landraces from Yunnan Province, China (uppercase N), and 51 other cultivars and landraces from other areas of China (uppercase YN). Another 41 accessions, numbered 400 through 440 (uppercase P, Additional file 2: Table S2), also from China, were used for genotyping of the *ALK* haplotype by AS-PCR molecular markers (Additional file 2: Table S1). The RDP1 accessions, provided by the International Rice Research Institute (IRRI), represent a global collection of accessions originating from over 60 rice growing countries and are publicly available from IRRI or the Genetic Stocks-*Oryza* (Eizenga et al. 2014). These accessions represent the five major rice subpopulations [*indica* (IND, 67 accessions), temperate *japonica* (TEJ, 43), tropical *japonica* (TRJ, 48), *aus* (AUS, 36) and *aromatic* (AROMATIC, 12)], a mixture of two or more subpopulations (ADMIX, 37) and an unclassified group (10), based on McCouch et al. (2016). The collection resources of N-numbered series are 109 cultivated rice from the Hani terraces area of Yunnan Province, with their subspopulation previously identified with 51 SSR markers (Li et al. 2017). The remaining 92 Chinese cultivars (identified as YN and P) were collected from the major rice production regions, and their subpopulation was determined with the same 51 SSR markers as reported by Li et al. (2017). Besides, two NILs, NIL(*ALK*^*b*^) and NIL(*ALK*^*c*^), were generated in the Nip background as described below. We state here that the accessions (Additional file 2: Table S2), the two NILs, the cultivars from the Hani terraces (Li et al. 2017) and the 92 diverse Chinese cultivars are available to Chinese researchers from the authors through a reasonable request. All rice accessions, cultivars and the two NILs were grown in the experimental fields at Yangzhou University, Jiangsu, China, or Linshui country, Hainan, China according to normal field management.

### DNA Isolation, Sequencing and Molecular Marker Development

Genomic DNA was isolated from young leaves following the method of Murray and Thompson (1980). A sequence of 4422 bp from the start codon (ATG) to the termination codon (TGA) was selected for amplification and subsequent sequencing in 399 accessions (No. 1–399 in Additional file 2: Table S2). Four pairs of primers, ALK-a_1_/ALK-a_2_, ALK-b_1_/ALK-b_2_, ALK-c_1_/ALK-c_2_, and ALK-d_1_/ALK-d_2_ (Additional file 2: Table S1), were used to amplify the genomic fragments. The PCR products were separated by electrophoresis using a 1% agarose gel and subsequently purified for sequencing. The AS-PCR markers, 4211 (G/A) and 4342 (GC/TT) (Fig. 1c–d; Additional file 2: Table S1), were designed using Primer Premier version 5.0 software and based on the SNPs in exon 8 among *ALK* alleles, which were used to select and detect the NILs (Additional file 1: Figure S2a). The above AS-PCR products were separated by 3% agarose gel electrophoresis.

### Generation of near-Isogenic Lines

Nip, containing the *ALK*^*a*^ allele with low GT and here named as Nip(*ALK*^*a*^), was used as the recurrent parent to generate the NILs. Two *indica* cultivars, 9311 and 9308 carrying the *ALK*^*b*^ and *ALK*^*c*^ alleles and showing low GT and high GT, respectively, were used as the donors during backcrossing (Additional file 1: Figure S2a). F_1_ plants were obtained by crossing of the donor and recurrent parent and were subsequently advanced up to the BC_7_ generation by MAS using the above AS-PCR molecular markers. Starting from the BC_1_F_1_, and in each of the following generations, approximately 60 plants were genotyped for the selected molecular markers. Among them, the individuals carrying the target *ALK* allele and phenotypically similar to the recurrent parent were selected for the next backcross until BC_7_F_1_. After several self-pollinations, the resulted NILs, NIL(*ALK*^*b*^) and NIL(*ALK*^*c*^), were then obtained for further experiments. To detect the genetic background in the NILs, the whole-genome resequencing method based on Zhang et al. (2011) was used. No off-target chromosomal segments from the donors 9311 or 9308 including any other grain quality-related genes were identified in the selected NILs (Additional file 1: Figure S3). The two NILs together with their recurrent parent (Nip) were grown to maturity in the experimental paddy field at Yangzhou University (Jiangsu, China) during the summer months (May to September, 2017) under natural conditions. The two NILs phenotypically had the same appearance as the Nip plants (Additional file 1: Figure S2b). All the conditions remained the same throughout the growing season. Mature seeds from 10 plants were harvested, air-dried, and used for grain quality analysis. Data for each sample is the mean value of three plots.

### qRT-PCR

Total RNA was extracted from developing endosperms of dehulled seeds harvested at 15 DAF. The first-strand cDNA used as the template, was synthesized by using a PrimeScript RT reagent kit (Takara, Kusatsu, Japan). The quantitative real-time reverse transcription PCR (qRT-PCR) was performed on the MyiQ real-time system (Bio-Rad) using AceQ q-PCR SYBR Green Master Mix (Vazyme). The rice *Actin1* gene was used as the internal control to normalize gene expression. The primers used for qRT-PCR are listed in Additional file 2: Table S1. The experiments were performed in triplicate for each sample.

### Rice Flour and Starch Preparation

Mature seeds were dehusked using a rice huller (model SY88-TH, Sangyong, Korea) and polished using a grain polisher (Kett, Tokyo, Japan). Milled rice samples were stored in airtight plastic bags in a refrigerator (4 °C) until further evaluation. The polished rice was ground into flour in a mill (FOSS 1093 Cyclotec Sample Mill, Sweden) through a 0.5 mm screen. Rice starch was isolated as described by Zhang et al. (2013).

### Determination of Gelatinization Properties

The GT was appraised using both the alkali digestion test and differential scanning calorimeter (DSC). For measurement of the alkali spreading value (ASV), four whole-grain, milled rice samples were placed in round plastic boxes containing 10 mL 1.4% potassium hydroxide (KOH). The boxes were incubated for 23 h at 30 °C. For DSC measurement, the rice flour over a 100-mesh screen was dried in a 40 °C oven for 2 days, balanced at room temperature for 2 days in a paper bag, and then sealed in a small plastic bag. The thermodynamic characteristics of the rice flour, including onset temperature (*T*_o_), peak temperature (*T*_p_), terminate temperature (*T*_c_), were tested using a DSC 200F_3_ thermal analyzer (Netzsch Instruments NA LLC, Burlington, MA, USA) according to the method of Zhang et al. (2013).

In addition, the gelatinization of rice flour in 4 M urea solution was measured as described by Satoh et al. (2003) with minor modifications. Briefly, 20 mg of rice flour mixed with 1 mL 4 M urea in a 1.5 mL centrifuge tube was incubated at 25 °C for 24 h. An aliquot (0.1 mL) of the solution was transferred to a new test tube with 5 mL of distilled water and then mixed well with 0.05 mL of 0.2% (w/v) iodide (I_2_) and 2% (w/v) potassium iodide (KI). The absorbance spectra of the solution were measured using an Infinite® 200 PRO multimode reader (Tecan Group Ltd., Seestrasse, Switzerland).

### Measurements of AAC, GC and RVA

The AAC was measured using iodine colorimetry (Juliano et al. 1981). The accurate AC was calculated based on GPC analysis (see below for details). The gel consistency (GC) was measured following the method of Tan et al. (1999). The pasting properties of rice starch were investigated using a Rapid Visco-Analyzer (RVA) (Techmaster, Newport Scientific, Warriewood, Australia) according to the methods of Zhu et al. (2012).

### Measurement of Starch Fine Structure

The relative molecular weight distribution of debranched starch was determined using gel permeation chromatograms (GPC) as described by Zhu et al. (2012). The GPC data used to draw the molecular weight distribution curves was transformed by integral equations based on standard dextran of known molecular weights. For comparison of amylopectin (AP), amylopectin short chain (AP1), amylopectin long chain (AP2), and amylose (AM), two replicate measurements were performed, and the analyses were normalized to have the same area under the curve (And and Corke 1999). The chain length distribution (CLD) of amylopectin was assessed using HPAEC and samples were prepared as described previously (Zhu et al. 2012). The crystalline structure of starches was measured by both XRD and Varian 7000 FTIR spectrometer system (ATR-FTIR) as described by Zhang et al. (2013). The FTIR absorbance values at 1047 and 1022 cm^− 1^ were extracted from spectra after correction. The above experiments were carried out with two technical replicates.

### Statistical Analysis

Three replicate measurements were performed unless otherwise specified. All data were represented as the means ± standard deviation. Data were subjected to one-way analysis of variance (ANOVA) and Tukey’s multiple comparison analysis using SPSS 16.0 statistical software program (IBM Corp, Armonk, NY, USA, https://www.ibm.com/products/spss-statistics). Results with a corresponding probability value of *P* < 0.05 were considered statistically significant.

## Supplementary information

**Additional file 1: Figure S1.** Distribution of polymorphisms of the 4422 bp sequences from the start codon (ATG) to the termination codon (TGA) within *ALK* gene and association analysis between these sequence polymorphisms and initial gelatinization temperature (*T*_o_). **Figure S2.** Construction of near isogenic lines (NILs) carrying different *ALK* alleles in Nipponbare (Nip) background. **Figure S3.** Physical maps of two near-isogenic lines (NILs) in Nipponbare (Nip) background based on the whole-genome resequencing data. **Figure S4.** The spectra patterns of rice starches revealed by (**a**) X-ray Diffraction (XRD) and (**b**) Attenuated Total Reflectance-Fourier Transform Infrared System (ATR-FTIR).

**Additional file 2: Table S1.** Primers used in this study. **Table S2.** Distributions of three main *ALK* haplotypes and thermo-properties determined by differential scanning calorimeter (DSC) among 440 rice accessions. **Table S3.** Distributions of three main *ALK* haplotypes from 440 rice accessions amongst the five rice subpopulations (*indica*, *aus*, *aromatic*, and temperate and tropical *japonica*) and subgroups (admixture and unclassified).

## Data Availability

The datasets supporting the conclusions of this article are provided within the article and its additional files. The RDP1 accessions are publicly available from IRRI or the Genetic Stocks-Oryza, and the other accessions and NILs are available from Qiaoquan Liu on a reasonable request.

## Abbreviations

AAC: Apparent amylose content
AC: Amylose content
AM: Amylose
AP: Amylopectin
AS-PCR: Allele-specific PCR
ASV: Alkali spreading value
BDV: Breakdown
CLD: Chain length distribution
CPV: Cool pasting viscosity
CSV: Consistence viscosity
DAF: Days after flowering
DP: Degree of polymerization
DSC: Differential scanning calorimeter
ECQ: Eating and cooking quality
FNP: Functional nucleotide polymorphism
GC: Gel consistency
GPC: Gel permeation chromatography
GT: Gelatinization temperature
HPAEC: High-performance anion-exchange chromatography
HPV: Hot peak viscosity
IR: Infrared ratio
MAS: Marker-assisted selection
NIL: Near-isogenic line
Nip: Nipponbare
PaT: Pasting temperature
PeT: Peak time
PKV: Peak viscosity
RVA: Rapid Visco Analyzer
SBV: Setback
SNP: Single nucleotide polymorphism
SSIIa: Soluble starch synthase IIa
*T*_c_: Terminate temperature of gelatinization
*T*_o_: Onset temperature of gelatinization
*T*_p_: Peak temperature of gelatinization
