# Supplementary material for: Genetic Dissection and Functional Differentiation of ALKa and ALKb, Two Natural Alleles of the ALK/SSIIa Gene, Responding to Low Gelatinization Temperature in Rice
Source: Rice (N Y). 2020 Jun 11;13:39. doi: 10.1186/s12284-020-00393-5 (PMC7289932; doi:10.1186/s12284-020-00393-5)
Supplement: Supplementary file 1 — Additional file 1: Figure S1. Distribution of polymorphisms of the 4422 bp sequences from the start codon (ATG) to the termination codon (TGA) within ALK gene and association analysis between these sequence polymorphisms and initial gelatinization temperature (To). Figure S2. Construction of near isogenic lines (NILs) carrying different ALK alleles in Nipponbare (Nip) background. Figure S3. Physical maps of two near-isogenic lines (NILs) in Nipponbare (Nip) background based on the whole-genome resequencing data. Figure S4. The spectra patterns of rice starches revealed by (a) X-ray Diffraction (XRD) and (b) Attenuated Total Reflectance-Fourier Transform Infrared System (ATR-FTIR). [file 12284_2020_393_MOESM1_ESM.docx]

**Additional file 1: Figure S1-S4**


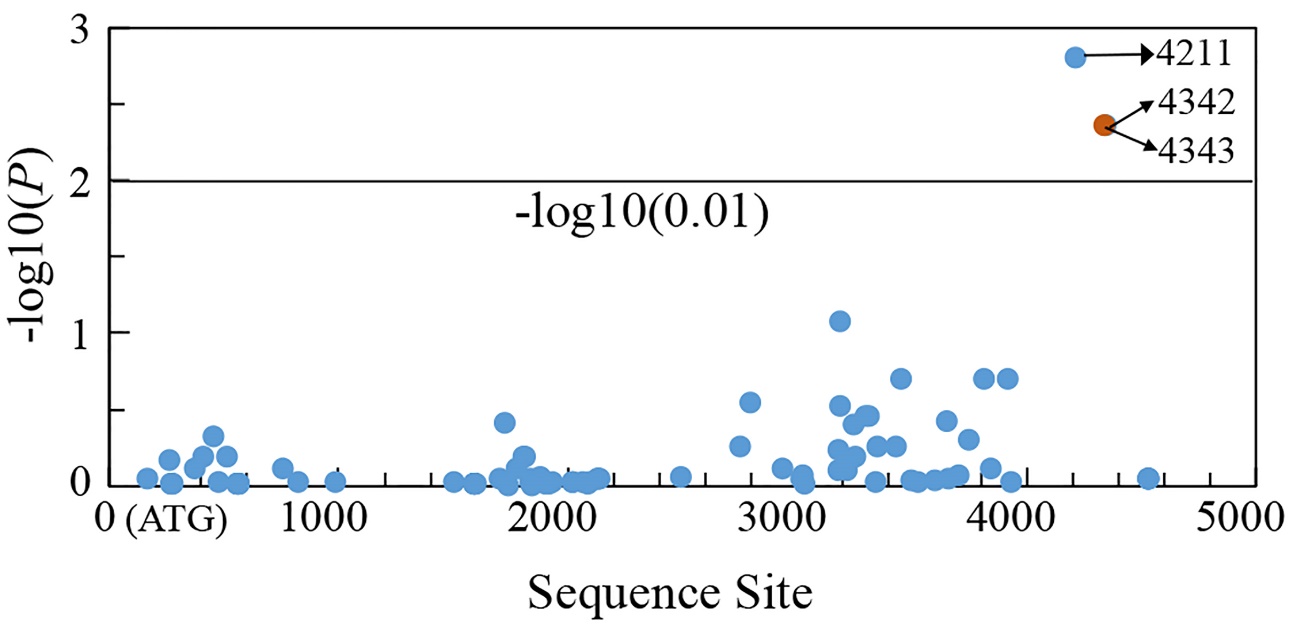


**Figure S1** Distribution of polymorphisms of the 4422 bp sequences from the start codon (ATG) to the termination codon (TGA) within *ALK* gene and association analysis between these sequence polymorphisms and onset gelatinization temperature (*T*_o_). Three SNPs (single nucleotide polymorphisms) in exon 8 as indicted by the arrows, 4211 (G/A), 4342 (G/T) and 4343 (C/T), respectively, were identified to be highly associated with gelatinization temperature in tested rice accessions.


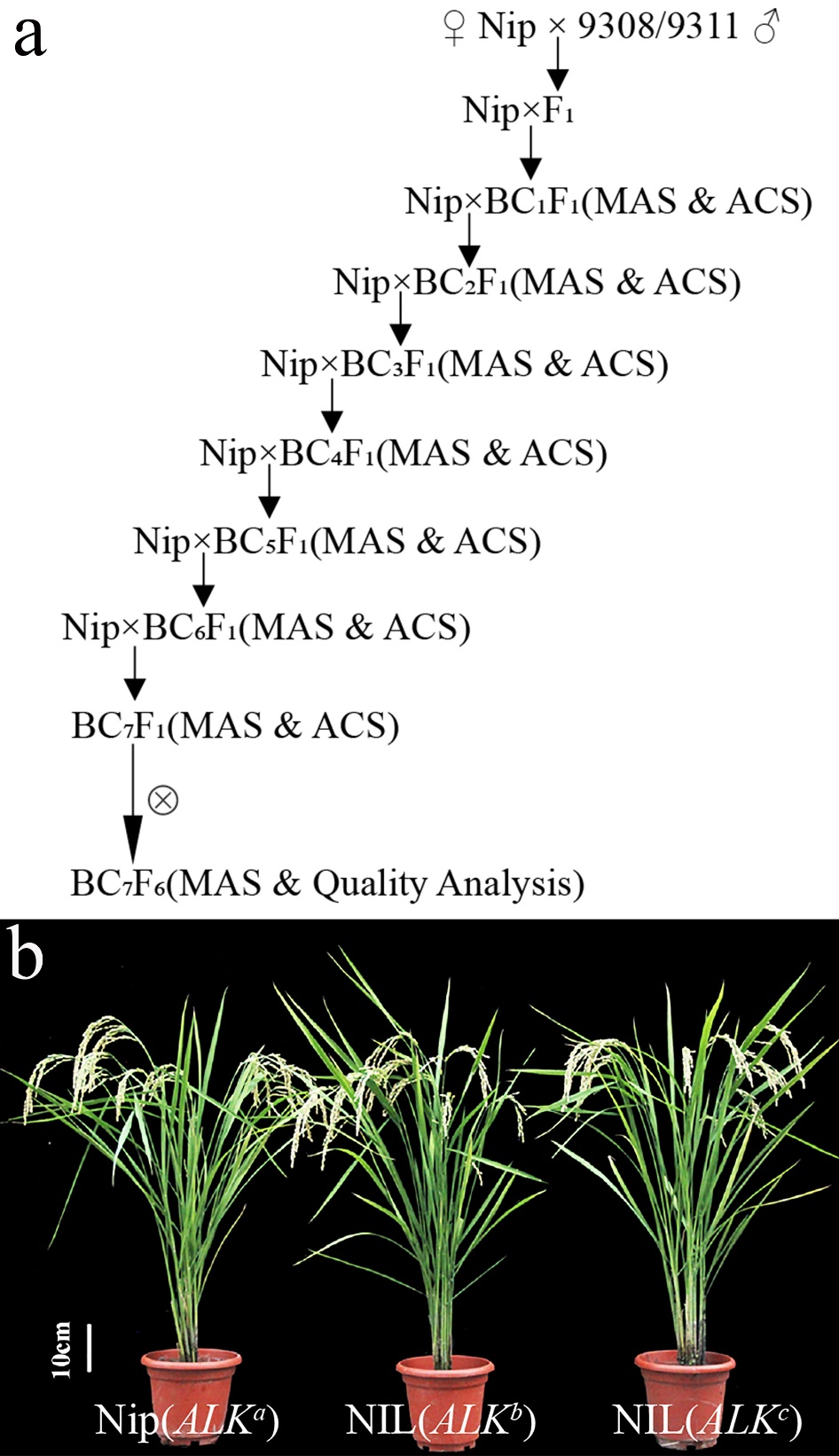


**Figure S2** Construction of near isogenic lines (NILs) carrying different *ALK* alleles in Nipponbare (Nip) background. **a** The flow chart for generation of NILs. **b** Plant morphology at the mature stage. Nip represents the *japonica* cultivar Nipponbare used as the recurrent parent carrying *ALK^a^* allele, while 9311 and 9308 are two *indica* cultivars used as the donors with *ALK^b^* and *ALK^c^* alleles, respectively. MAS, molecular marker-assisted selection; ACS, agronomic character selection.


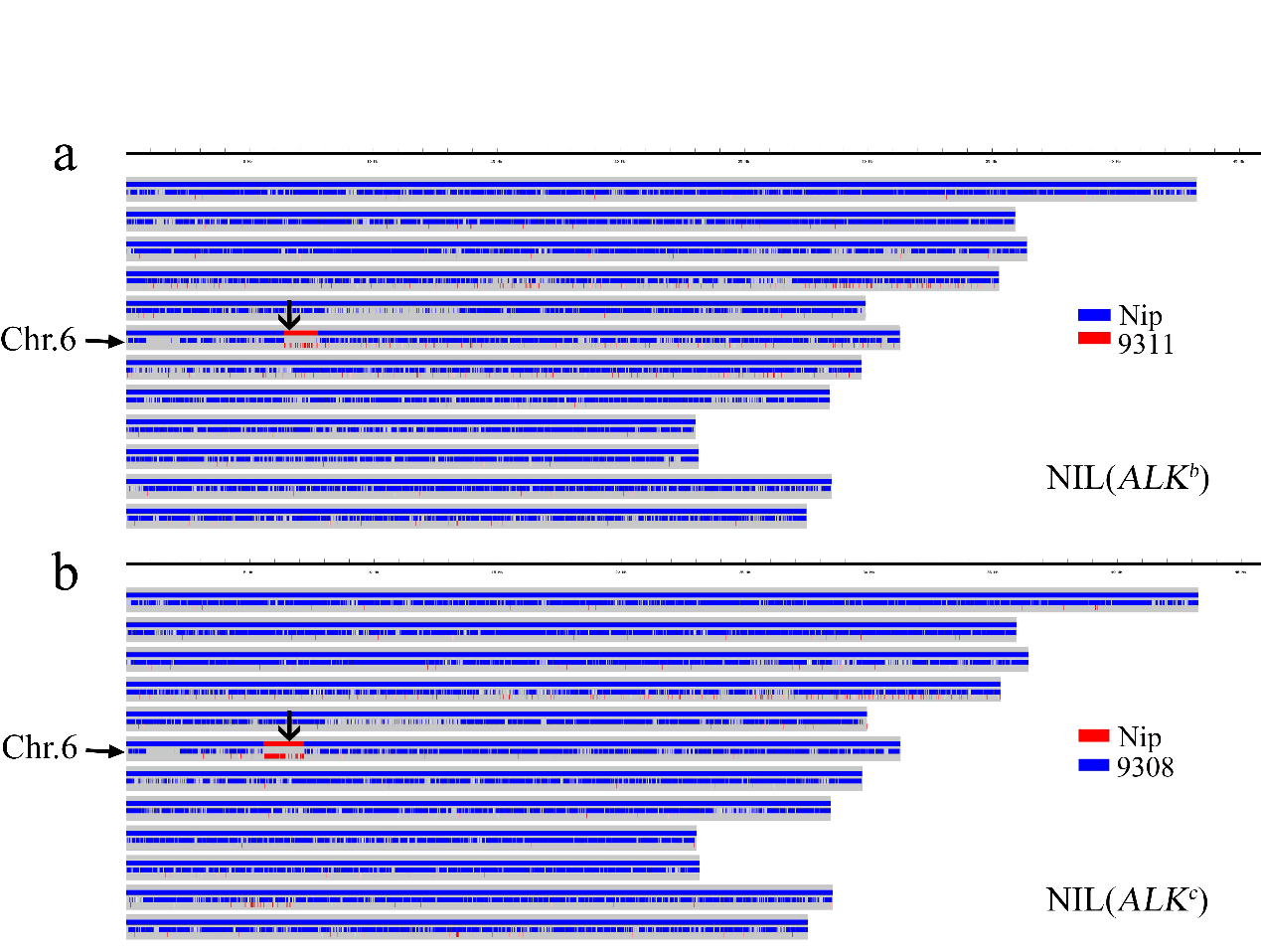


**Figure S3** Physical maps of two near-isogenic lines (NILs) in Nipponbare (Nip) background based on the whole-genome resequencing data. **a** and **b** indicate the NIL(*ALK^b^*) and NIL(*ALK^c^*) with *ALK^b^* or *ALK^c^* allele, respectively. The rows represent 12 rice chromosomes, respectively. The red areas indicate the substituted chromosome segments (containing *ALK* locus) from the two donors, *indica* cultivars 9311 (**a**) and 9308 (**b**), respectively, while the blue areas indicate chromosome regions of *japonica* Nip.


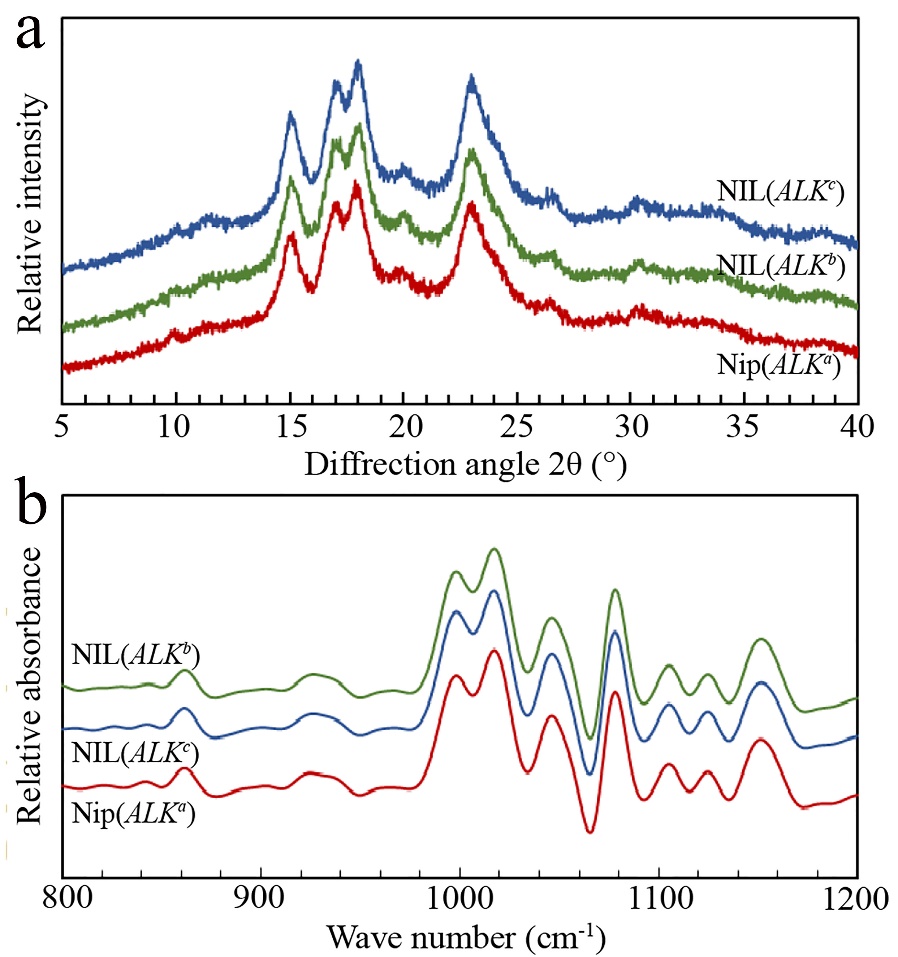


**Figure S4** The spectra patterns of rice starches revealed by (**a**) X-ray Diffraction (XRD) and (**b**) Attenuated Total Reflectance-Fourier Transform Infrared System (ATR-FTIR). Nip(*ALK^a^*), NIL(*ALK^b^*) and NIL(*ALK^c^*) are three near-isogenic lines (NILs) carrying different *ALK* alleles in Nipponbare (Nip) background.
